# Thickness bound for nonlocal wide-field-of-view metalenses

**DOI:** 10.1038/s41377-022-01038-6

**Published:** 2022-12-01

**Authors:** Shiyu Li, Chia Wei Hsu

**Affiliations:** Ming Hsieh Department of Electrical and Computer Engineering, University of Southern California, Los Angeles, CA 90089 USA

**Keywords:** Metamaterials, Photonic devices, Nanophotonics and plasmonics

## Abstract

Metalenses—flat lenses made with optical metasurfaces—promise to enable thinner, cheaper, and better imaging systems. Achieving a sufficient angular field of view (FOV) is crucial toward that goal and requires a tailored incident-angle-dependent response. Here, we show that there is an intrinsic trade-off between achieving a desired broad-angle response and reducing the thickness of the device. Like the memory effect in disordered media, this thickness bound originates from the Fourier transform duality between space and angle. One can write down the transmission matrix describing the desired angle-dependent response, convert it to the spatial basis where its degree of nonlocality can be quantified through a lateral spreading, and determine the minimal device thickness based on such a required lateral spreading. This approach is general. When applied to wide-FOV lenses, it predicts the minimal thickness as a function of the FOV, lens diameter, and numerical aperture. The bound is tight, as some inverse-designed multi-layer metasurfaces can approach the minimal thickness we found. This work offers guidance for the design of nonlocal metasurfaces, proposes a new framework for establishing bounds, and reveals the relation between angular diversity and spatial footprint in multi-channel systems.

## Abstract (web summary)

A transmission-matrix-based framework is proposed to establish the intrinsic link between angular diversity and spatial footprint in multi-channel systems and to derive a bound on the thickness of nonlocal metasurfaces.

## Graphical abstract

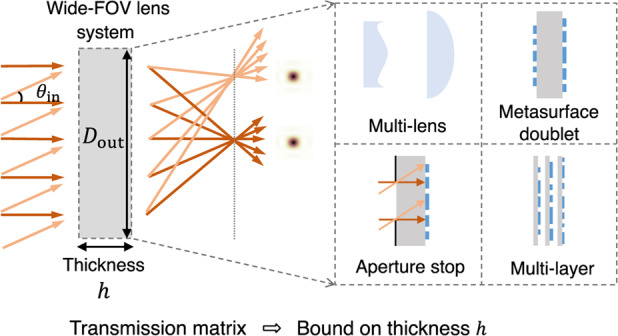

## Introduction

**Fig. 1:**
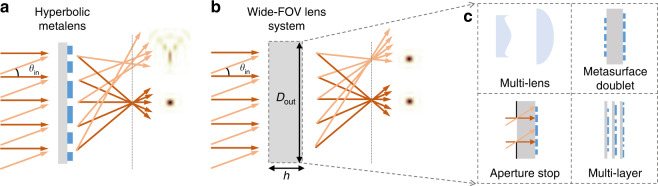
Wide-FOV lens systems. Schematics of **a** a metalens with a hyperbolic phase profile and **b** a diffraction-limited lens system with a wide FOV. The former can have subwavelength thickness and produces a diffraction-limited focal spot at normal incidence but suffers from strong aberrations at oblique incidence. The latter achieves diffraction-limited focusing over a wide range of incident angles but requires a minimal thickness *h*. **c** Examples of systems that realize wide-FOV diffraction-limited focusing: cascade of multiple lenses, metasurface doublets, use of an aperture stop, and multi-layer metasurfaces

Metasurfaces use subwavelength building blocks to achieve versatile functions with spatially-resolved modulation of the phase, amplitude, and polarization of light^1–10^. Among them, metalenses^11–15^ receive great attention given their potential to enable thinner, lighter, cheaper, and better imaging systems for a wide range of applications where miniaturization is critical (e.g. for bio-imaging and endoscopy and for mobile and wearable devices such as cell phones and mixed-reality headsets). Metalenses are commonly modeled by a spatially-varying transmission phase-shift profile $\phi (x,y)$ where *x*, *y* are the transverse coordinates. To focus normal-incident light to a diffraction-limited spot with focal length *f*, one can require all of the transmitted light to be in phase when reaching the focal spot, which gives a hyperbolic phase profile^16,17^

1 $$\phi _{{{{\mathrm{hyp}}}}}(x,y) = \frac{{2\pi }}{\lambda }\left( {f - \sqrt {f^2 + x^2 + y^2} } \right)$$

where *λ* is the operating wavelength. However, for oblique illumination, the optical path lengths of the marginal rays no longer match that of the chief ray, resulting in coma, astigmatism, and field-curvature aberrations^18–20^ as schematically illustrated in Fig. 1a. These aberrations severely limit the input angular range over which focusing is achieved (i.e., the FOV).

One way to expand the FOV is to use the phase profile of an equivalent spherical lens^14^ or a quadratic phase profile^21–23^, which reduce off-axis aberrations. However, doing so introduces spherical aberration and defocus aberration, with a reduced effective aperture size, axial elongation, and a low Strehl ratio^14,23,24^, so the focus is no longer diffraction-limited.

To achieve wide FOV with diffraction-limited focusing, one can use metasurface doublets^25–32^ or triplets^33^ analogous to conventional multi-lens systems, add an aperture stop so incident light from different angles reach different regions of the metasurface^34–39^, or use inverse-designed multi-layer structures^40,41^; these approaches are schematically illustrated in Fig. 1b, c. Notably, all of these approaches involve a much thicker system where the overall thickness (*e*.*g*., separation between the aperture stop and the metasurface) plays a critical role. Meanwhile, miniaturization is an important consideration and motivation for metalenses. This points to the scientifically and technologically important questions: is there a fundamental trade-off between the FOV and the thickness of a metalens system, or lenses in general? If so, what is the minimal thickness allowed by physical laws?

Light propagating through disordered media exhibits an angular correlation called “memory effect” ^42–46^: when the incident angle tilts, the transmitted wavefront stays invariant and tilts by the same amount if the input momentum tilt is smaller than roughly one over the medium thickness. Weakly scattering media like a diffuser exhibit a longer memory effect range^47^, and thin layers like a metasurface also have a long memory effects range^48^. With angle-multiplexed volume holograms, it was found that a thicker hologram material is needed to store more pages of information at different angles^49,50^. These phenomena suggest there may be an intrinsic relation between angular diversity and thickness in multi-channel systems including but not limited to lenses.

Bounds for metasurfaces can provide valuable physical insights and guidance for future designs. Shrestha et al.^51^ and Presutti et al.^52^ related the maximal operational bandwidth of achromatic metalenses to the numerical aperture (NA), lens diameter, and thickness, which was generalized to wide-FOV operation by Shastri et al.^53^ and diffractive lenses by Engelberg et al.^54^. Shastri et al. investigated the relation between absorber efficiency and its omnidirectionality^55^, Gigli et al. analyzed the limitations of Huygens’ metasurfaces due to nonlocal interactions^56^, Chung et al. determined the upper bounds on the efficiencies of unit-cell-based high-NA metalenses^57^, Yang et al. quantified the relation between optical performance and design parameters for aperture-stop-based metalenses^39^, and Martins et al. studied the trade-off between the resolution and FOV for doublet-based metalenses^32^. Each of these studies concerns one specific type of design. The power-concentration bound of Zhang et al. ^58^ and the multifunctional bound of Shim et al. ^59^ are more general, though they bound the performance rather than the device footprint. However, the relationship between thickness and angular diversity remains unknown.

In this work, we establish such relationship and apply it to wide-FOV metalenses. Given any desired angle-dependent response, we can write down its transmission matrix, measure its degree of nonlocality (as encapsulated in the lateral spreading of incident waves encoded in the transmission matrix), from which we determine the minimal device thickness. This is a new approach for establishing bounds, applicable across different designs including single-layer metasurfaces, cascaded metasurfaces, diffractive lenses, bulk metamaterials, thick volumetric structures, *etc*.

## Results

### Thickness bound via transmission matrix

The multi-channel transport through any linear system can be described by a transmission matrix. Consider monochromatic wave at angular frequency $\omega = 2\pi c/\lambda$. The incoming wavefront can be written as a superposition of propagating waves at different angles and polarizations, as

2 $${{{\mathbf{E}}}}_{{{{\mathrm{in}}}}}\left( {{{{\boldsymbol{\rho }}}},z = 0} \right) = \mathop {\sum }\limits_{a = 1}^{N_{{{{\mathrm{in}}}}}} v_a\hat e_ae^{i{{{\mathbf{k}}}}_\parallel ^a \cdot {{{\boldsymbol{\rho }}}}}w_{{{{\mathrm{in}}}}}\left( {{{\boldsymbol{\rho }}}} \right)$$

where ${{{\boldsymbol{\rho }}}} = \left( {x,y} \right)$ is the transverse coordinate; $\hat e_a$ and ${{{\mathbf{k}}}}_\parallel ^a = ( {k_x^a,k_y^a} )$ are the polarization state and the transverse wave number (momentum) of the *a*-th plane-wave input with amplitude $v_a$; *z* = *0* is the front surface of the lens, and $w_{{{{\mathrm{in}}}}}\left( {{{\boldsymbol{\rho }}}} \right) = 1$ for $\left| {{{\boldsymbol{\rho }}}} \right| < D_{{{{\mathrm{in}}}}}/2$ (zero otherwise) is a window function that describes an aperture that blocks incident light beyond entrance diameter *D*_in_. The wave number ${{{\mathbf{k}}}}_\parallel ^a$ is restricted to propagating waves within the angular FOV, with $\left| {{{{\mathbf{k}}}}_\parallel ^a} \right| < \left( {\omega /c} \right)\sin \left( {{{{\mathrm{FOV}}}}/2} \right)$. Since the input is band-limited in space due to the entrance aperture, a discrete sampling of ${{{\mathbf{k}}}}_\parallel ^a$ with $2\pi /D_{{{{\mathrm{in}}}}}$ spacing at the Nyquist rate^60^ is sufficient. Therefore, the number *N*_in_ of “input channels” is finite^61^, and the incident wavefront is parameterized by a column vector ${{{\boldsymbol{v}}}} = \left[ {v_1, \cdots ,v_{N_{{{{\mathrm{in}}}}}}} \right]^{{{\mathrm{T}}}}$. Similarly, the propagating part of the transmitted wave is a superposition of *N*_out_ output channels at different angles and polarizations,

3 $${{{\mathbf{E}}}}_{{{\mathrm{t}}}}\left( {{{{\boldsymbol{\rho }}}},z = h} \right) = \mathop {\sum }\limits_{b = 1}^{N_{{{{\mathrm{out}}}}}} u_b\hat e_be^{i{{{\mathbf{k}}}}_\parallel ^b \cdot {{{\boldsymbol{\rho }}}}}w_{{{{\mathrm{out}}}}}\left( {{{\boldsymbol{\rho }}}} \right)$$

where *h* is the thickness of the lens system, and the window function $w_{{{{\mathrm{out}}}}}\left( {{{\boldsymbol{\rho }}}} \right) = 1$ for $\left| {{{\boldsymbol{\rho }}}} \right| < D_{{{{\mathrm{out}}}}}/2$ blocks transmitted light beyond an output aperture with diameter *D*_out_. The transmitted wavefront is parameterized by column vector ${{{\boldsymbol{u}}}} = \left[ {u_1, \cdots ,u_{N_{{{{\mathrm{out}}}}}}} \right]^{{{\mathrm{T}}}}$. Normalization prefactors are ignored in Eqs. (2)–(3) for simplicity.

The input and the output must be related through a linear transformation, so we can write

4 $$u_b = \mathop {\sum }\limits_{a = 1}^{N_{{{{\mathrm{in}}}}}} t_{ba}v_a$$

or ${{{\boldsymbol{u}}}} = {{{\mathbf{t}}}}{{{\boldsymbol{v}}}}$, where **t** is the transmission matrix^62–64^. The transmission matrix describes the exact wave transport through any linear system, regardless of the complexity of the structure and its material compositions.

**Fig. 2:**
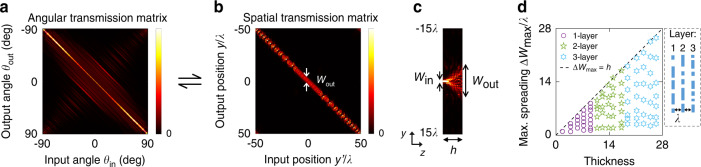
Transmission matrix and its relation to nonlocality and device thickness *h*. The same transmission matrix (**a**) in angular basis $\left| {t\left( {k_y,k_y^\prime } \right)} \right|^2$ and (**b**) in spatial basis $\left| {t\left( {y,y^\prime } \right)} \right|^2$, for a hyperbolic metalens with diameter $D = 100\lambda$, ${{{\mathrm{NA}}}} = 0.45$, thickness $h = 4.2\lambda$, index contrast = 0.4, without a substrate ($n_{{{{\mathrm{in}}}}} = 1$). The axes in (**a**) are linearly spaced in $k_y = \left( {\omega /c} \right)\sin \theta _{{{{\mathrm{out}}}}}$ and $k_y^\prime = \left( {\omega /c} \right)\sin \theta _{{{{\mathrm{in}}}}}$. **c** Intensity profile inside the metasurface for a localized input at $y^\prime = 0$, corresponding to the middle column of the spatial transmission matrix. The lateral spreading $\Delta W = W_{{{{\mathrm{out}}}}} - W_{{{{\mathrm{in}}}}}$ quantifies the degree of nonlocality. **d** Maximal lateral spreading $\Delta W_{{{{\mathrm{max}}}}} \equiv \mathop {{\max }}\nolimits_{y^\prime } \Delta W\left( {y^\prime } \right)$ computed from $t\left( {y,y^\prime } \right)$, for random metasurfaces with varying thickness and varying number of layers at FOV = 180° ($W_{{{{\mathrm{in}}}}} = 0.75\lambda$). The inset shows a schematic of the multi-layer structures. The data reveal an empirical inequality $\Delta W_{{{{\mathrm{max}}}}} < h$, which places a lower bound on the thickness of a device that realizes the corresponding transmission matrix

For simplicity, in the examples below we consider the transverse magnetic (TM) waves of 2D systems where we only need to consider the $\hat x$ polarization ${{{\mathbf{E}}}} = E_x\left( {y,z} \right)\hat x$, with the transverse coordinate *ρ* *=* *y* and the transverse momentum *k*_*y*_ both being scalars. We compute the transmission matrix with full-wave simulations using the recently proposed augmented partial factorization method^24^ implemented in the open-source software MESTI^65^. Figure 2a shows the squared amplitude of the transmission matrix for a 2D metalens designed to exhibit the hyperbolic phase profile in Eq. (1) at normal incidence. We informally express such transmission matrix in angular basis as $t\left( {k_y,k_y^\prime } \right)$ where $k_y^\prime = k_y^a = \left( {\omega /c} \right)\sin \theta _{{{{\mathrm{in}}}}}$ is the transverse momentum of the input and $k_y = k_y^b = \left( {\omega /c} \right)\sin \theta _{{{{\mathrm{out}}}}}$ is that of the output.

Each windowed plane-wave input or output is itself a superposition of spatially-localized waves, so we can convert the transmission matrix from the angular basis to a spatial basis with no change in its information content. Informally, such a change of basis is described by a Fourier transform *F* on the input side and an inverse Fourier transform $F^{ - 1}$ on the output side^66^, as

5 $$t\left( {y,y^\prime } \right) = F^{ - 1}t\left( {k_y,k_y^\prime } \right)F$$

A formal derivation is provided in the Supplementary Materials. Intuitively, $t\left( {y,y^\prime } \right)$ gives the output at position *y* given a localized incident wave focused at $y^\prime$; it has also been called the “discrete-space impulse response”^67^. Figure 2b shows the transmission matrix of Fig. 2a in spatial basis. The output profile is approximately the same near the lens center because the hyperbolic metalens can be treated as a linear space-invariant system under paraxial approximation.

The off-diagonal elements of $t\left( {y,y^\prime } \right)$ capture nonlocal couplings between different elements of a metasurface, which are commonly ignored in conventional metasurface designs but play a critical role for angular diversity because of the Fourier transform duality between space and angle. To gain intuition, consider another Fourier dual between frequency and time: a dispersive medium has a frequency-dependent response, and a short pulse (localized in time *t*′ because its frequency components are in phase) propagating through such dispersive medium necessarily gets stretched into a longer pulse (less localized in time *t* because its frequency components are no longer in phase). Analogously, here if a metasurface has an angle-dependent response, an incident wave localized at $y^\prime = y_0$ (with its angular components $k_y^\prime$ in phase at $y^\prime = y_0$) propagating through such metasurface must spread and become less localized in *y* (as its angular components *k*_*y*_ are no longer in phase at $y = y_0$). More angular diversity necessitates more lateral spreading (*i*.*e*., more nonlocality).

Such nonlocal spreading links to the system thickness *h*. Given a thicker device, incident light at *z* = *0* can spread more laterally when it reaches the other side at *z* *=* *h* due to diffraction. We define the lateral spreading ∆*W* as the difference between the width of the output and that of the input,

6 $$\Delta W\left( {y^\prime } \right) = W_{{{{\mathrm{out}}}}}\left( {y^\prime } \right) - W_{{{{\mathrm{in}}}}}$$

as indicated in Fig. 2c on a numerically computed intensity profile with a localized incident wave. The output width $W_{{{{\mathrm{out}}}}}$ is also the vertical width of the near-diagonal elements of the spatial transmission matrix $t\left( {y,y^\prime } \right)$, as indicated in Fig. 2b.

To quantify the transverse widths, we use the inverse participation ratio (IPR)^68^, with

7 $$W_{{{{\mathrm{out}}}}}\left( {y^\prime } \right) = \frac{{\left[ {{\int} {\left| {t\left( {y,y^\prime } \right)} \right|} ^2dy} \right]^2}}{{{\int} {\left| {t\left( {y,y^\prime } \right)} \right|} ^4dy}}$$

For rectangular functions, the IPR equals the width of the function. The width of the input is similarly defined: in the spatial basis, each input consists of plane waves with momenta $\left| {k_y^\prime } \right| < \left( {2\pi /\lambda } \right)\sin \left( {{{{\mathrm{FOV}}}}/2} \right)$ that make up a sinc profile in space: $E_{{{{\mathrm{in}}}}}\left( {y^\prime } \right) \propto {{{\mathrm{sinc}}}}\left( {k_{y^\prime }^{{{{\mathrm{max}}}}}y^\prime } \right)$ with $k_{y^\prime }^{{{{\mathrm{max}}}}} = \left( {2\pi /\lambda } \right)\sin \left( {{{{\mathrm{FOV}}}}/2} \right)$, whose IPR is $W_{{{{\mathrm{in}}}}} = 3\lambda /\left[ {4\sin \left( {{{{\mathrm{FOV}}}}/2} \right)} \right]$.

The nonlocal lateral spreading $\Delta W\left( {y^\prime } \right)$ depends on the location $y^\prime$ of illumination. Since we want to relate lateral spreading to the device footprint which is typically measured by the thickness at its thickest part, below we will consider the maximal lateral spreading across the surface,

8 $$\Delta W_{{{{\mathrm{max}}}}} \equiv \mathop{\max}\limits_{y^\prime }\Delta W\left( {y^\prime } \right)$$

Figure 2d shows the maximal spreading $\Delta W_{{{{\mathrm{max}}}}}$ as a function of thickness *h*, calculated from full-wave simulations using MESTI^65^. Here we consider metasurfaces with random phase profiles and different number of layers. Each layer has identical thickness and is separated by distance *λ*. These data points cover NA from 0.1 to 0.9, index contrasts from 0.1 to 2, using diameter $D = 100\lambda$, with the full ${{{\mathrm{FOV}}}} = 180^\circ$ and thus $W_{{{{\mathrm{in}}}}} = 0.75\lambda$. From these data, we observe an empirical inequality

9 $$\Delta W_{{{{\mathrm{max}}}}}\, < \,h$$

as intuitively expected. This relation provides a quantitative link between the angle-dependent response of a system and its thickness.

Note that while higher index contrasts allow a *2π* phase shift to be realized with thinner metasurfaces, such higher index contrasts do not lower the minimum thickness governed by Eq. (9). The systems considered in Fig. 2d consider random metasurfaces under TM polarization, with no substrate, and use the full FOV; Figures S1–S3 in the Supplementary Materials further show that Eq. (9) also holds for metasurfaces under transverse-electric (TE) polarization, with the hyperbolic phase profile of Eq. (1) at normal incidence, with a quadratic phase profile^21–23^ at normal incidence, sitting on a substrate or with a reduced FOV (*i*.*e*. increased $W_{{{{\mathrm{in}}}}}$).

While we use 2D systems above to illustrate the concept, this transmission-matrix-based approach for establishing thickness bound readily applies to systems in 3D. In 3D, one would include the additional dimension and both polarizations in the transmission matrix, apply two-dimensional Fourier transforms in Eq. (5), compute the characteristic input/output areas through the IPR, and obtain the lateral spreading from the diameters of the input/output areas. The computations are more involved, but the steps are the same as in 2D. Intuitively, we expect a relation similar to Eq. (9) in 3D (likely with a slightly different prefactor).

We emphasize that even though Eq. (9) follows intuition and is found to be valid across a wide range of systems considered above, it remains empirical. In particular, in the presence of guided resonances^69,70^, it is possible for the incident wave from free space to be partially converted to a guided wave and then radiate out to the free space after some in-plane propagation, enabling the lateral spreading *∆W* to exceed the thickness *h*; this is likely the case with resonance-based space-squeezing systems^71–73^. Indeed, we have found that Eq. (9) may be violated within a narrow angular range near that of a guided resonance. It is possible to extend the angular range by stacking multiple resonances^73^ or by using guided resonances on a flat band^74,75^, but doing so restricts the degrees of freedom for further designs. In the following, we assume Eq. (9) is valid, which implicitly excludes broad-angle resonant effects.

Given the angle-dependent response of a system described by $t\left( {k_y,k_y^\prime } \right)$, Eqs. (5)–(9) quantify its degree of nonlocality and the minimal thickness such a system must have. This formalism applies to different nonlocal systems. Below, we use this formalism to establish a thickness bound for wide-FOV lenses.

### Thickness bound for wide-FOV lenses

#### Transmission matrix of an ideal wide-FOV lens

**Fig. 3:**
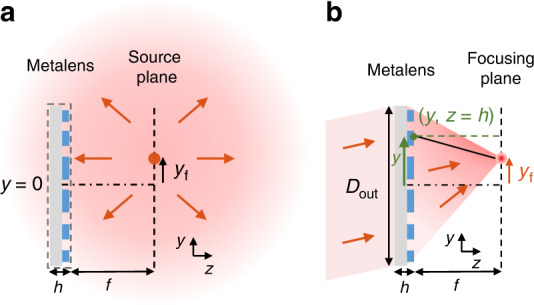
Schematics for determining the field transmitted through an ideal metalens. **a** Outgoing field from a point source located at the focal spot $\left( {y = y_{{{\mathrm{f}}}},z = h + f} \right)$. **b** To ideally focus light to such focal spot, the transmitted field across the back aperture $D_{{{{\mathrm{out}}}}}$ should be proportional to the complex conjugation of the radiated field in (**a**), given by the distance $r = \sqrt {f^2 + \left( {y - y_{{{\mathrm{f}}}}} \right)^2}$ between a point (*y,h*) on the back of the metalens to the focal spot $\left( {y_{{{\mathrm{f}}}},h + f} \right)$

To ideally focus a windowed (within $\left| {y^\prime } \right|\, <\, D_{{{{\mathrm{in}}}}}/2$) plane wave $E_x^a\left( {y^\prime ,z = 0} \right) = E_0e^{ik_y^\prime y^\prime }$ incident from angle $\theta _{{{{\mathrm{in}}}}}$ to point ${{{\mathbf{r}}}}_{{{\mathrm{f}}}}\left( {\theta _{{{{\mathrm{in}}}}}} \right) = \left( {y = y_{{{\mathrm{f}}}}\left( {\theta _{{{{\mathrm{in}}}}}} \right),z = h + f} \right)$ on the focal plane, the field on the back surface of a metalens should be proportional to the conjugation of the field radiated from a point source at the focal spot to the back surface, as illustrated in Fig. 3. Here we consider such ideal transmitted field across the entire back aperture of the lens within $\left| y \right| < D_{{{{\mathrm{out}}}}}/2$, independent of the incident angle. Note that the angular distribution of the output depends on the incident angle, so the lens is not telecentric. The radiated field from a point source in 2D is proportional to $e^{ikr}/\sqrt r$, and the distance is $r = \sqrt {f^2 + \left( {y - y_{{{\mathrm{f}}}}} \right)^2}$, so the ideal field on the back surface of a metalens is

10 $$E_x^a\left( {y,z = h} \right) = \left\{ {\begin{array}{*{20}{l}} {A\left( {\theta _{{{{\mathrm{in}}}}}} \right)\frac{{e^{i\phi _{{{{\mathrm{out}}}}}^{{{{\mathrm{ideal}}}}}\left( {y,\theta _{{{{\mathrm{in}}}}}} \right)}}}{{\left[ {f^2 + \left( {y - y_{{{\mathrm{f}}}}} \right)^2} \right]^{1/4}}}{\mathrm{for}}\left| y \right| < \frac{{D_{{{{\mathrm{out}}}}}}}{2}} \\ {0 \qquad\qquad\qquad\qquad\; {\mathrm{otherwise}}} \end{array}} \right.$$

where $A\left( {\theta _{{{{\mathrm{in}}}}}} \right)$ is a constant amplitude, and the ideal phase distribution on the back of the metalens is^11,19,35^

11 $$\phi _{{{{\mathrm{out}}}}}^{{{{\mathrm{ideal}}}}}\left( {y,\theta _{{{{\mathrm{in}}}}}} \right) = \psi \left( {\theta _{{{{\mathrm{in}}}}}} \right) - \frac{{2\pi }}{\lambda }\sqrt {f^2 + \left[ {y - y_{{{\mathrm{f}}}}\left( {\theta _{{{{\mathrm{in}}}}}} \right)} \right]^2}$$

A global phase does not affect focusing, so we include a spatially-constant (but can be angle-dependent) phase function $\psi \left( {\theta _{{{{\mathrm{in}}}}}} \right)$. For the focal spot position, we consider $y_{{{\mathrm{f}}}}\left( {\theta _{{{{\mathrm{in}}}}}} \right) = f\tan \theta _{{{{\mathrm{in}}}}}$, such that the chief ray going through the lens center remains straight. A lens system that realizes this angle-dependent phase shift profile $\Delta \phi _{{{{\mathrm{ideal}}}}}\left( {y,\theta _{{{{\mathrm{in}}}}}} \right) = \phi _{{{{\mathrm{out}}}}}^{{{{\mathrm{ideal}}}}}\left( {y,\theta _{{{{\mathrm{in}}}}}} \right) - \phi _{{{{\mathrm{in}}}}}\left( {y,\theta _{{{{\mathrm{in}}}}}} \right)$ within the desired $\left| {\theta _{{{{\mathrm{in}}}}}} \right| \,< \,{{{\mathrm{FOV}}}}/2$ will achieve diffraction-limited focusing with no aberration, where $\phi _{{{{\mathrm{in}}}}}\left( {y,\theta _{{{{\mathrm{in}}}}}} \right) = \left( {\omega /c} \right)\sin \theta _{{{{\mathrm{in}}}}}\,y$ is the phase profile of the incident light.

We project the ideal output field in Eq. (10) onto a set of flux-orthogonal windowed plane-wave basis to get the angular transmission matrix $t\left( {k_y,k_y^\prime } \right)$, as

12 $$t_{ba} = \sqrt {\frac{{k_z^b}}{{D_{{{{\mathrm{out}}}}}}}} \mathop {\int }\limits_{ - \frac{{D_{{{{\mathrm{out}}}}}}}{2}}^{\frac{{D_{{{{\mathrm{out}}}}}}}{2}} E_x^a\left( {y,z = h} \right)e^{ - ik_y^by}dy$$

where $k_y^a = a\left( {2\pi /D_{{{{\mathrm{in}}}}}} \right)$ with $a \in {\mathbb{Z}}$ and $| {k_y^a} | < ( {\omega /c} )\sin ( {{{{\mathrm{FOV}}}}/2} )$, $k_y^b = b\left( {2\pi /D_{{{{\mathrm{out}}}}}} \right)$ with $b \in {\mathbb{Z}}$ and $| {k_y^b} | < \omega /c$, and $( {k_y^a} )^2 + ( {k_z^a} )^2 = ( {k_y^b} )^2 + ( {k_z^b} )^2 = ( {\omega /c} )^2$. The spatial transmission matrix $t\left( {y,y^\prime } \right)$ is then given by

13 $$t\left( {y,y^\prime } \right) = \frac{1}{{\sqrt {D_{{{{\mathrm{in}}}}}D_{{{{\mathrm{out}}}}}} }}\mathop {\sum }\limits_b \mathop {\sum }\limits_a \sqrt {\frac{{k_z^a}}{{k_z^b}}} e^{ik_y^by}t_{ba}e^{ - ik_y^ay^\prime }$$

where $\left| y \right| < D_{{{{\mathrm{out}}}}}/2$ and $\left| {y^\prime } \right| < D_{{{{\mathrm{in}}}}}/2$. Detailed derivations and implementations of Eqs. (12)–(13) are given in Supplementary Sec. 2. From $t\left( {y,y^\prime } \right)$, we obtain the lateral spreading $\Delta W\left( {y{^\prime} } \right)$.

#### Thickness bound

**Fig. 4:**
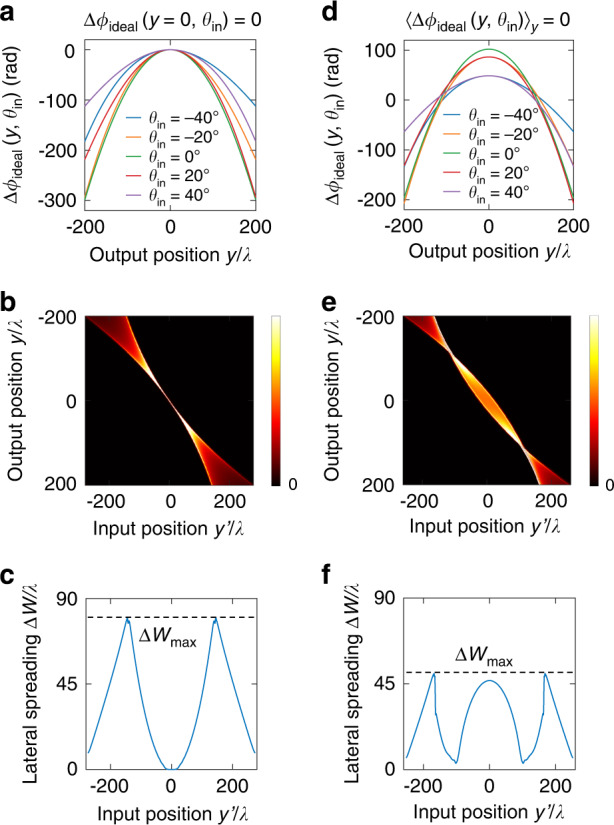
Angle-dependent phase shift and lateral spreading of an ideal large-FOV lens. **a**–**c** The incident-angle-dependent phase-shift profiles, spatial transmission matrix $\left| {t\left( {y,y^\prime } \right)} \right|^2$, and lateral spreading $\Delta W\left( {y^\prime } \right)$ respectively for an ideal large-FOV lens with the global phase $\psi \left( {\theta _{{{{\mathrm{in}}}}}} \right)$ chosen such that $\Delta \phi _{{{{\mathrm{ideal}}}}}\left( {y = 0,\theta _{{{{\mathrm{in}}}}}} \right) = 0$; this choice minimizes the angle dependence of the phase shift at $y = 0$, which minimizes $\Delta W\left( {y^\prime = 0} \right)$. **d**–**f** Corresponding plots with $\psi \left( {\theta _{{{{\mathrm{in}}}}}} \right) = \psi _0\left( {\theta _{{{{\mathrm{in}}}}}} \right)$ in Eq. (15), chosen such that $\left\langle {\Delta \phi _{{{{\mathrm{ideal}}}}}\left( {y,\theta _{{{{\mathrm{in}}}}}} \right)} \right\rangle _y = 0$ which minimizes $\Delta W_{{{{\mathrm{max}}}}}$ and therefore minimizes the thickness bound. Lens parameters: diameter $D_{{{{\mathrm{out}}}}} = 400\lambda$, NA = 0.45, ${{{\mathrm{FOV}}}} = 80^\circ$, with $y_{{{\mathrm{f}}}}\left( {\theta _{{{{\mathrm{in}}}}}} \right) = f\tan \theta _{{{{\mathrm{in}}}}}$

Figure 4a–c plots $\Delta \phi _{{{{\mathrm{ideal}}}}}\left( {y,\theta _{{{{\mathrm{in}}}}}} \right)$, the corresponding transmission matrix $t\left( {y,y^\prime } \right)$ in spatial basis, and $\Delta W\left( {y^\prime } \right)$ for a lens with output diameter $D_{{{{\mathrm{out}}}}} = 400\lambda$, ${{{\mathrm{NA}}}} = \sin \left( {{{{\mathrm{arctan}}}}\left( {D_{{{{\mathrm{out}}}}}/\left( {2f} \right)} \right)} \right) = 0.45$ (NA is defined based on normal incidence), ${{{\mathrm{FOV}}}} = 80^\circ$. Here, the global phase $\psi \left( {\theta _{{{{\mathrm{in}}}}}} \right) = \frac{{2\pi }}{\lambda }\sqrt {f^2 + y_{{{\mathrm{f}}}}\left( {\theta _{{{{\mathrm{in}}}}}} \right)^2}$ is chosen such that $\Delta \phi _{{{{\mathrm{ideal}}}}}\left( {y = 0,\theta _{{{{\mathrm{in}}}}}} \right) = 0$. Note that unlike in Fig. 2b, here $\Delta W\left( {y^\prime } \right)$ depends strongly on the position $y^\prime$. An input focused at $y^\prime = 0$ is a superposition of plane waves with different angles that constructively interfere at $y^\prime = 0$, and since the phase shift $\Delta \phi _{{{{\mathrm{ideal}}}}}\left( {y = 0,\theta _{{{{\mathrm{in}}}}}} \right) = 0$ is angle-independent there, the transmitted plane waves at different angles still interfere constructively at the output *y* = *0*, with no lateral spreading, so $\Delta W\left( {y^\prime = 0} \right) \approx 0$. However, away from the lens center, the phase shift $\Delta \phi _{{{{\mathrm{ideal}}}}}\left( {y \,\ne\, 0,\theta _{{{{\mathrm{in}}}}}} \right)$ exhibits strong angle dependence as shown in Fig. 4a, resulting in significant lateral spreading as shown in Fig. 4b, c.

In the above example, $\Delta W_{{{{\mathrm{max}}}}} \equiv \mathop {{\max }}\limits_{y^\prime } \Delta W\left( {y^\prime } \right) \approx 80\lambda$. Through Eq. (9), we can then conclude that such a lens must be at least 80*λ* thick, regardless of how the lens is designed. This 80*λ* is the axial distance light must propagate in order to accumulate the desired angle-dependent phase shift and the associated lateral spreading. Recall that ∆*W* is also a measure of nonlocality, so the unavoidable lateral spreading here indicates that aberration-free wide-FOV lenses must be nonlocal.

This example uses one particular global phase function $\psi \left( {\theta _{{{{\mathrm{in}}}}}} \right) = \frac{{2\pi }}{\lambda }\sqrt {f^2 + y_{{{\mathrm{f}}}}\left( {\theta _{{{{\mathrm{in}}}}}} \right)^2}$. Different $\psi \left( {\theta _{{{{\mathrm{in}}}}}} \right)$ lead to different phase shifts $\Delta \phi _{{{{\mathrm{ideal}}}}}\left( {y,\theta _{{{{\mathrm{in}}}}}} \right) = \phi _{{{{\mathrm{out}}}}}^{{{{\mathrm{ideal}}}}}\left( {y,\theta _{{{{\mathrm{in}}}}}} \right) - \phi _{{{{\mathrm{in}}}}}\left( {y,\theta _{{{{\mathrm{in}}}}}} \right)$, with different $\Delta W_{{{{\mathrm{max}}}}}$ and different minimal thickness. Since $\psi \left( {\theta _{{{{\mathrm{in}}}}}} \right)$ does not affect the focusing quality, we can further lower the thickness bound by optimizing over $\psi \left( {\theta _{{{{\mathrm{in}}}}}} \right)$ as follows.

#### Minimization of maximal spreading

To minimize $\Delta W_{{{{\mathrm{max}}}}}$ and the resulting thickness bound, we search for the function $\psi \left( {\theta _{{{{\mathrm{in}}}}}} \right)$ that minimizes the maximal phase-shift difference among all possible pairs of incident angles across the whole surface,

14 $$\mathop {{{{{\mathrm{argmin}}}}}}\limits_{\psi \left( {\theta _{{{{\mathrm{in}}}}}} \right)} \mathop {{\max }}\limits_{y,\theta _{{{{\mathrm{in}}}}}^i,\theta _{{{{\mathrm{in}}}}}^j} \left| {\Delta \phi _{{{{\mathrm{ideal}}}}}\left( {y,\theta _{{{{\mathrm{in}}}}}^i;\psi } \right) - \Delta \phi _{{{{\mathrm{ideal}}}}}\left( {y,\theta _{{{{\mathrm{in}}}}}^j;\psi } \right)} \right|^2$$

where $$\left| y \right| < D_{{{{\mathrm{out}}}}}/2$$ and $$\left| {\theta _{{{{\mathrm{in}}}}}^{i,j}} \right| < {{{\mathrm{FOV}}}}/2$$.

A sensible choice is $\psi \left( {\theta _{{{{\mathrm{in}}}}}} \right) = \psi _0\left( {\theta _{{{{\mathrm{in}}}}}} \right)$ with

15 $$\psi _0\left( {\theta _{{{{\mathrm{in}}}}}} \right) = \frac{{2\pi }}{\lambda }\left\langle {\sqrt {f^2 + \left[ {y - y_{{{\mathrm{f}}}}\left( {\theta _{{{{\mathrm{in}}}}}} \right)} \right]^2} + y\sin \theta _{{{{\mathrm{in}}}}}} \right\rangle _y$$

where $\left\langle { \cdots } \right\rangle_y$ denotes averaging over *y* within $\left| y \right| < D_{{{{\mathrm{out}}}}}/2$. With this choice, the phase profiles at different incident angles are all centered around the same *y*-averaged phase, namely $\left\langle {\Delta \phi _{{{{\mathrm{ideal}}}}}\left( {y,\theta _{{{{\mathrm{in}}}}}} \right)} \right\rangle _y = 0$ for all $\theta _{{{{\mathrm{in}}}}}$, so the worst-case variation with respect to $\theta _{{{{\mathrm{in}}}}}$ is reduced. Figure 4d–f shows the resulting phase profile, spatial transmission matrix, and $\Delta W\left( {y^\prime } \right)$ with this $\psi = \psi _0$. Indeed, we observe $\Delta W_{{{{\mathrm{max}}}}}$ to lower from $80\lambda$ to $50\lambda$ compared to the choice of $\Delta \phi _{{{{\mathrm{ideal}}}}}\left( {y = 0,\theta _{{{{\mathrm{in}}}}}} \right) = 0$ in Fig. 4c.

Eq. (14) is a convex problem^76^, so its global minimum can be found with established algorithms. We use the CVX package^77,78^ to perform this convex optimization. Section 3 and Fig. S6 of Supplementary Materials show that the $\psi _0\left( {\theta _{{{{\mathrm{in}}}}}} \right)$ in Eq. (15) is very close to the global optimum of Eq. (14), and the two give almost identical $\Delta W_{{{{\mathrm{max}}}}}$. Therefore, in the following we adopt the $\psi _0\left( {\theta _{{{{\mathrm{in}}}}}} \right)$ in Eq. (15) to obtain the smallest-possible thickness bound.

One can potentially also vary the focal spot position $y_{{{\mathrm{f}}}}\left( {\theta _{{{{\mathrm{in}}}}}} \right)$ to further minimize $\Delta W_{{{{\mathrm{max}}}}}$, since image distortions can be corrected by software. After optimizing over $y_{{{\mathrm{f}}}}$, we find that $y_{{{\mathrm{f}}}}\left( {\theta _{{{{\mathrm{in}}}}}} \right) = f\tan \theta _{{{{\mathrm{in}}}}}$ already provides close-to-minimal $\Delta W_{{{{\mathrm{max}}}}}$.

#### Dependence on lens parameters

The above procedure can be applied to any wide-FOV lens. For example, we now know that the lens considered in Fig. 4 must be at least 50*λ* thick regardless of its design. It is helpful to also know how such a minimal thickness depends on the lens parameters, so we carry out a systematic study here.

Supplementary Video 1 shows how the ideal transmission matrix in both bases evolve as the FOV increases. While increasing the FOV only adds more columns to the angular transmission matrix, doing so increases the variation of the phase shift with respect to the incident angle (*i*.*e*., increases the angular diversity), which changes the spatial transmission matrix and increases the lateral spreading (*i*.*e*., increases nonlocality). An analogy using the time-frequency Fourier pair is that when a pulse propagates through a dispersive medium, increasing the spectral bandwidth makes the input pulse shorter but with more pulse stretching during propagation because the output spectral phase is misaligned over a larger bandwidth. We also observe that the output profiles in $\left| {t\left( {y,y^\prime } \right)} \right|^2$ develop two strong peaks at the edges as the FOV increases. The IPR in Eq. (7) is better suited for functions that are unimodal or close to rectangular. Therefore, when ${{{\mathrm{FOV}}}} \ge 100^\circ$, we use the full width at half maximum (FWHM) instead to quantify $W_{{{{\mathrm{out}}}}}$; Figure S8 of the Supplementary Materials shows that the FWHM equals IPR for small FOV but is a better measure of the output width for large FOV.

**Fig. 5:**
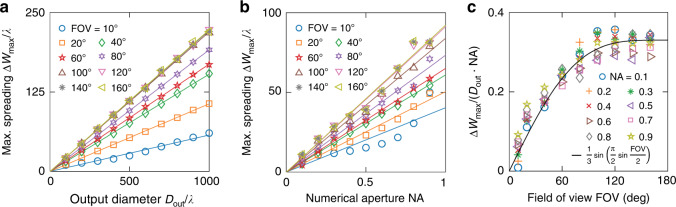
Dependence of the optimized maximal lateral spreading $\Delta W_{{{{\mathrm{max}}}}}$ on the parameters of an aberration-free wide-FOV lens. **a** $\Delta W_{{{{\mathrm{max}}}}}$ as a function of the output diameter $D_{{{{\mathrm{out}}}}}$ when NA = 0.7. **b** $\Delta W_{{{{\mathrm{max}}}}}$ as a function of the numerical aperture NA when $D_{{{{\mathrm{out}}}}} = 300\lambda$. Symbols are the maximal spreading of such lenses, and solid lines are linear fits. **c** $\Delta W_{{{{\mathrm{max}}}}}/\left( {D_{{{{\mathrm{out}}}}} \cdot {{{\mathrm{NA}}}}} \right)$ as a function of the FOV. Black solid line is Eq. (16)

Next, we quantify the dependence on all lens parameters. Figure 5 plots the optimized maximal lateral spreading $\Delta W_{{{{\mathrm{max}}}}}$ as a function of the output diameter $D_{{{{\mathrm{out}}}}}$, NA and the FOV. As shown in Fig. 5a, $\Delta W_{{{{\mathrm{max}}}}}$ grows linearly with $D_{{{{\mathrm{out}}}}}$ for different FOV. Figure 5b further shows that $\Delta W_{{{{\mathrm{max}}}}}$ also grows approximately linearly with the numerical aperture NA. Figure 5a, b fixes NA = 0.7 and $D_{{{{\mathrm{out}}}}} = 300\lambda$ respectively, while similar dependencies are observed for other lens parameters (Figs. S9–10 of Supplementary Materials). Dividing by $D_{{{{\mathrm{out}}}}}$ and NA, we obtain how $\Delta W_{{{{\mathrm{max}}}}}$ depends on the FOV, shown in Fig. 5c. The angular range is governed by $\sin \left( {{{{\mathrm{FOV}}}}/2} \right)$, but the functional dependence of $\Delta W_{{{{\mathrm{max}}}}}$ on the FOV is not simply $\sin \left( {{{{\mathrm{FOV}}}}/2} \right)$; empirically, we find the function $\frac{1}{3}\sin \left( {\frac{\pi }{2}\sin \frac{{{{{\mathrm{FOV}}}}}}{2}} \right)$ to provide a reasonable fit for the FOV dependence. These dependencies can be summarized as

16 $$\Delta W_{{{{\mathrm{max}}}}} \approx \left( {\frac{1}{3}{{{\mathrm{NA}}}}} \right)D_{{{{\mathrm{out}}}}}\sin \left( {\frac{\pi }{2}\sin \frac{{{{{\mathrm{FOV}}}}}}{2}} \right)$$

Equation (9) and Eq. (16) then tell us approximately how the thickness bound varies with the lens parameters,

17 $$h \gtrsim \left( {\frac{1}{3}{{{\mathrm{NA}}}}} \right)D_{{{{\mathrm{out}}}}}\sin \left( {\frac{\pi }{2}\sin \frac{{{{{\mathrm{FOV}}}}}}{2}} \right)$$

This result applies to both TM and TE polarizations. It makes intuitive sense, since increasing the NA, aperture size, and/or FOV will all lead to an increased phase-shift variation, which leads to the increased minimal thickness. Equation (17) also shows that imaging systems with a larger space-bandwidth product necessarily require a larger thickness.

Any aberration-free wide-FOV lens system must have a transmission matrix, so the above bound applies to any such system regardless of how the system is designed (barring unlikely broad-angle resonant effects). This result shows that to achieve large FOV with a wide output aperture, a single layer of subwavelength-thick metasurface is fundamentally not sufficient. Meanwhile, it also reveals room to make existing designs more compact, as we discuss below.

While the results above are obtained for 2D systems, we expect qualitatively similar results in 3D (likely with a different prefactor) since the relation between angular diversity and lateral spreading and the relation between lateral spreading and thickness are both generic. Note that we use FOV to denote the range of incident angles from air. Equation (17) continues to hold in the presence of substrates, with the Snell’s law $\sin \frac{{{{{\mathrm{FOV}}}}}}{2} = n_{{{{\mathrm{in}}}}}\sin \frac{{{{{\mathrm{FOV}}}}_{{{{\mathrm{in}}}}}}}{2}$ for ${{{\mathrm{FOV}}}}_{{{{\mathrm{in}}}}}$ in a substrate with refractive index $n_{{{{\mathrm{in}}}}}$, since we have shown in Fig. S2 that Eq. (9) holds in the presence of a substrate and since the ideal transmission matrix is the same with or without a substrate.

Table 1 lists diffraction-limited wide-FOV metalens systems reported in the literature. All of them have total thickness consistent with Eq. (17). A few inverse-designed multi-layer structures^40,41^ have thickness close to the bound, suggesting that the bound is tight. Note that the second design in ref. ^41^ has a slightly smaller thickness (24*λ*) than the bound (25*λ*), likely because it only optimizes for diffraction-limited focusing at a discrete set of angles. Existing metalenses based on doublets or aperture stops are substantially thicker than the bound, which is sensible since those systems have ample amount of free spaces not used for structural design.

**Table 1:** Metalenses with diffraction-limited focusing over a wide FOV^1^

	Method	Exp./Sim.	Output diameter $D_{{{{\mathrm{out}}}}}(D_{{{{\mathrm{out}}}}}^{{{{\mathrm{eff}}}}})$	Numerical aperture	FOV (air)	Strehl ratio	Total thickness	Thickness bound
Arbabi et al.^25^	Doublet	3D Exp.	(800 μm)	0.49	_60°_	$\gtrsim 0.9$	1 mm	92 μm
Groever et al.^26^	Doublet	3D Exp.	(313 μm)	0.44	_50°_	$\gtrsim 0.8$	500 μm	30 μm
He et al.^27^	Doublet	3D Sim.	(400 μm)	0.47	_60°_	–	500 μm	44 μm
Li et al.^28^	Doublet	3D Sim.	(20 μm)	0.45	_50°_	$\gtrsim 0.5$	31.2 μm	1.8 μm
Tang et al.^29^	Doublet	3D Sim.	(30 μm)	0.35	_40°_	–	21.2 μm	1.8 μm
Kim et al.^30^	Doublet	3D Sim.	(300 μm)	0.38	_60°_	–	500 μm	27 μm
Huang et al.^31^	Doublet	3D Sim.	(5 μm)	0.60	_60°_	–	6.6 μm	0.7 μm
Engelberg et al.^34^	Aperture	3D Exp.	(1.35 mm)	0.20	_30°_	–	3.36 mm	0.03 mm
Shalaginov et al.^35^.	Aperture	3D Exp.	(1 mm)	0.24	_∼180°_	$\gtrsim 0.8$	2 mm	0.08 mm
Shalaginov et al.^35^	Aperture	3D Sim.	(1 mm)	0.20	_∼180°_	$\gtrsim 0.8$	3.9 mm	0.07 mm
Fan et al.^36^	Aperture	3D Sim.	(20 μm)	0.25	_170°_	$\gtrsim 0.8$	38.6 μm	1.7 μm
Zhang et al.^37^	Aperture	3D Exp.	(1 mm)	0.11	_∼180°_	–	5.44 mm	0.04 mm
Yang et al.^38^	Aperture	3D Sim.	(100 μm)	0.18	_∼180°_	~0.64	200 μm	6 μm
Lin et al.^40^	Multi-layer	2D Sim.	23*λ*	0.35	_40°_	–	1.5*λ*	1.4*λ*
Lin et al.^41^	Multi-layer	2D Sim.	50*λ*	0.24	_60°_	$\gtrsim 0.8$	12*λ*	2.8*λ*
Lin et al.^41^	Multi-layer	2D Sim.	125*λ*	0.70	_80°_	$\gtrsim 0.8$	24*λ*	25*λ*
Lin et al.^41^	Multi-layer	3D Sim.	50*λ*	0.12	_16°_	$\gtrsim 0.8$	12*λ*	0.4*λ*

^1^We note that the thickness bound here is directly from Eq. (17), which is an approximate expression and is obtained for 2D systems but suffices as an estimation. References^25–31,34–38^ adopt a telecentric configuration where each incident angle fills an effective diameter $D_{{{{\mathrm{out}}}}}^{{{{\mathrm{eff}}}}}$ within the output aperture, which we use in place of $D_{{{{\mathrm{out}}}}}$ when evaluating their thickness bounds. Some works also correct the chromatic aberration: at 473 nm and 532 nm in ref. ^29^, at 445 nm, 532 nm and 660 nm in ref. ^30^, from 470 nm to 650 nm in ref. ^31^, and from 1 to 1.2 μm in ref. ^38^. Reference^40^ achieves diffraction-limited focusing for 7 angles within the FOV. Reference^41^ achieves diffraction-limited focusing for 19, 7 and 9 angles within the FOV and also corrects the chromatic aberration for 10, 4, and 5 frequencies within a 23% spectral bandwidth from up to down.

Here we consider ideal aberration-free focusing for all incident angles within the FOV. Relaxing some of these conditions can relax the thickness bound; for example, if diffraction- limited focusing is not necessary, the quadratic phase profile^21–23^ can eliminate the angle dependence of the phase profile. Meanwhile, achromatic wide-FOV lenses^29–31,33,38,41^ will be subject to additional constraints beyond nonlocality^53^.

## Discussion

Due to the Fourier-transform duality between space and momentum, any multi-channel system with an angle-dependent response will necessarily require nonlocality and spatial spreading (exemplified in Fig. 4 and analogous to a pulse propagating through a dispersive medium under time-frequency duality), which is tied to the device thickness through Eq. (9). This relationship is not limited to wide-FOV lenses and establishes the intrinsic link between angular diversity and spatial footprint suggested in the introduction.

For example, one can readily use this approach to establish thickness bounds for other types of nonlocal metasurfaces such as retroreflectors^79^ and photovoltaic concentrators^40,80–82^ where a wide angular range is also desirable. Note that concentrators are additionally subject to efficiency bounds arising from passivity and/or reciprocity^58^.

These results can guide the design of future nonlocal metasurfaces, providing realistic targets for device dimensions. While multi-layer metasurfaces that reach Eq. (17) have not been experimentally realized yet, there are several realistic routes. A stacked triple-layer metalens has been reported^33^. Multi-layer structures have been realized with two-photon polymerization^82–84^, or repeated deposition and patterning of 2D layers^85–88^. Volumetric nanostructures may also be realized with deposition onto shrinking scaffolds^89^. Additionally, multi-level diffractive lenses can readily have thickness above 10 μm^90,91^.

Fundamental bounds like this are valuable as metasurface research evolves beyond single-layer local designs, as better control of light is achieved over wider ranges of angles, and with the continued push toward ultra-compact photonic devices. Future work can investigate designs incorporating broad-angle resonant responses. We also note that the transmission-matrix approach is versatile and can be used to establish other types of bounds beyond the device footprint.

## Materials and methods

Calculations for Fig. 4, Fig. 5, and Figs. S5–S10 are done by implementing Eqs. (10)–(15) in the main text and Eq. (S13), Eq. (S16), and Eqs. (S17)–(S19) in the Supplementary Materials.

For the full-wave simulations of Fig. 2, and Figs. S1-S3, we use the open-source software MESTI to obtain the angular transmission matrix of different types of metasurfaces and the intensity profile inside the metasurface. Two-dimensional metasurfaces with different diameters, phase profiles and NA are designed using a library of ridges with a periodicity of 0.4*λ* that can cover a phase-shift range of 2*π*. Different phase-shift values are realized by changing the widths of ridges. The simulation domain is discretized to 20 pixels per wavelength in the material with the highest refractive index, and is surrounded by 20 pixels of perfectly matched layers to attenuate the outgoing waves with sufficiently small reflection. More information about how to use MESTI to get the response of unit cells, design metasurfaces with certain phase distributions, and obtain their transmission matrices can be found in the examples of Ref. ^65^.

## Supplementary information

Supplementary information for Thickness bound for nonlocal wide-field-of-view metalenses

FOV dependence

## Data Availability

All data needed to evaluate the conclusions in this study are presented in the paper and in the supplementary materials.
