# Supplementary material for: Thickness bound for nonlocal wide-field-of-view metalenses
Source: Light Sci Appl. 2022 Dec 1;11:338. doi: 10.1038/s41377-022-01038-6 (PMC9715731; doi:10.1038/s41377-022-01038-6)
Supplement: Supplementary file 1 — Supplementary information for Thickness bound for nonlocal wide-field-of-view metalenses [file 41377_2022_1038_MOESM1_ESM.pdf]

Supplementary Information for
Thickness bound for nonlocal wide-field-of-view metalenses

Shiyu Li[1] and Chia Wei Hsu[1,*]

[1]Ming Hsieh Department of Electrical and Computer Engineering,
University of Southern California, Los Angeles, California 90089, USA

[*] Corresponding author: cwhsu@usc.edu

Supplementary Materials of
Thickness bound for nonlocal wide-field-of-view metalenses

This document provides supplemental information to "Thickness bound for nonlocal wide-field-of-view metalenses". It consists of six sections. Section 1 provides data of the lateral spreading as a function of thickness for metasurfaces under transverse-electric (TE) polarization, on a substrate or with different field of view (FOV). Section 2 details the definition of the transmission matrix in angular basis and in spatial basis. Section 3 describes the optimization of the global phase function $\psi(\theta_{\rm in})$ to reduce the angular variation of the phase shift and the maximal lateral spreading $\Delta W_{\rm max}$. In Sec. 4, we use an animation to show how the ideal transmission matrix in both bases and the phase-shift profiles evolve as the FOV increases. In Sec. 5, we show the cross sections of the spatial transmission matrix for ideal lenses with different FOV in comparison to the output widths $W_{\rm out}$ defined by the inverse participation ratio (IPR). In Sec. 6, we provide complete data plots for the optimized $\Delta W_{\rm max}$ as a function of the lens parameters.

1. LATERAL SPREADING VERSUS THICKNESS

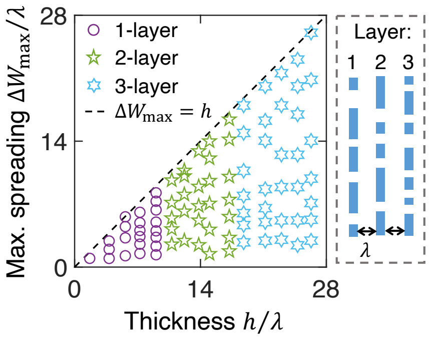

Fig. S1. Maximal lateral spreading $\Delta W_{\rm max}$ computed from the spatial transmission matrix, for random metasurfaces with varying thicknesses and varying number of layers under TE polarization at FOV = 180° (input width $W_{\rm in} = 0.75\lambda$). The inset shows a schematic of the multi-layer structures.

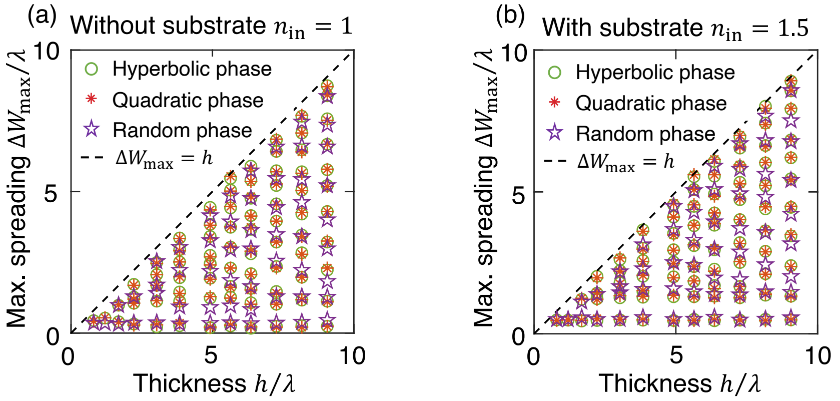

Fig. S2. Maximal lateral spreading $\Delta W_{\rm max}$ computed from the spatial transmission matrix, for different single-layer metasurfaces of varying thicknesses without ($n_{\rm in} = 1$) and with a substrate ($n_{\rm in} = 1.5$) at FOV = 180° (input width $W_{\rm in} = 0.75\lambda$).

Figure 2(d) of the main text plots the maximal lateral spreading $\Delta W_{\rm max}$ as a function of the thickness h for random metasurfaces with different number of layers and no substrate ($n_{\rm in} = 1$) under transverse-magnetic (TM) polarization at FOV = 180°. Figure S1 shows $\Delta W_{\rm max}$ of the same metasurfaces under TE-polarized incidence. Figure S2 shows $\Delta W_{\rm max}$ of single-layer metasurfaces designed to have the hyperbolic phase profile [1, 2] at normal incidence, quadratic phase profile [3–5] at normal incidence, and random phase profiles without ($n_{\rm in} = 1$) and with substrate ($n_{\rm in} = 1.5$) at FOV = 180°. We observe the same relation here: $\Delta W_{\rm max} < h$. This relation also holds when different FOV is considered, as shown in Fig. S3.

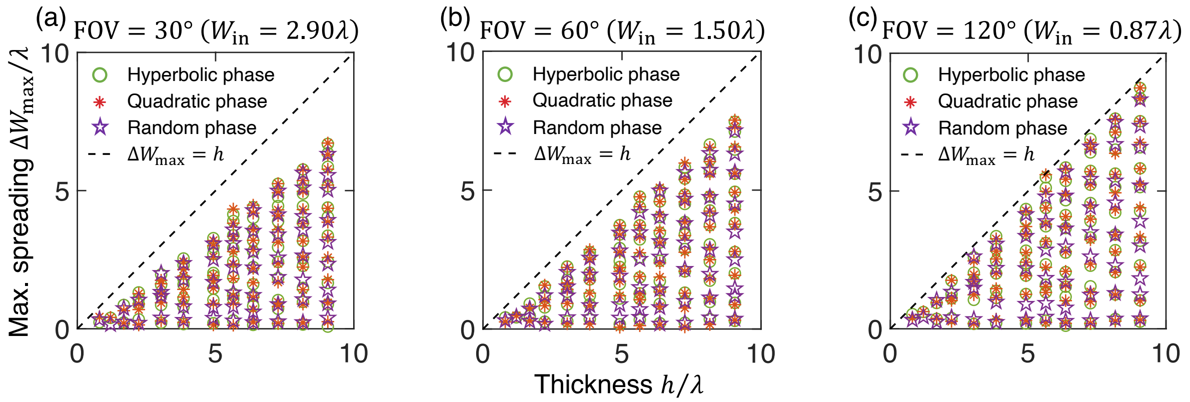

Fig. S3. Maximal lateral spreading $\Delta W_{\rm max}$ computed from the spatial transmission matrix, for different single-layer metasurfaces of varying thicknesses with no substrate ($n_{\rm in} = 1$) at smaller values of FOV, corresponding to input widths $W_{\rm in} = 2.90\lambda$, 1.50λ and 0.87λ from left to right.

2. TRANSMISSION MATRIX OF IDEAL WIDE-FOV METALENSES

A. Basis of transverse channels

Consider the TM fields of a 2D system, with $\mathbf{E} = E_x(y,z)\hat{x}$, where the refractive index is $n_{\rm in}$ and $n_{\rm out}$ on the incident ($z < 0$) and transmitted ($z > h$) sides respectively, as shown in Fig. S4. The Poynting flux for a monochromatic wave at ω is

$$\mathbf{S} = \frac{1}{2}\mathrm{Re}[\mathbf{E}^* \times \mathbf{H}] = \frac{1}{2\omega\mu_0}\mathrm{Im}\left(0, E_x^*\frac{\partial E_x}{\partial y}, E_x^*\frac{\partial E_x}{\partial z}\right). \tag{S1}$$

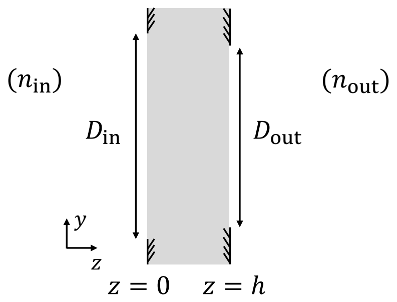

Fig. S4. Schematic of the system: light incident from a medium with refractive index $n_{\rm in}$ onto an entrance aperture with diameter $D_{\rm in}$, propagates through the structure with thickness h, and exits through an output aperture with diameter $D_{\rm out}$ into a medium with refractive index $n_{\rm out}$.

We expand the incident field near the input aperture, $E_x^{\rm in}(y, z \lesssim 0)$, in a basis of truncated plane waves:

$$E_x^{\rm in}(y, z \lesssim 0) = \sum_a v_a f_a(y,z), \tag{S2}$$

where

$$f_a(y,z) = \begin{cases} \frac{1}{\sqrt{D_{\rm in}}}\frac{1}{\sqrt{k_z^a}}e^{i(k_y^a y + k_z^a z)} & \text{for } |y| < \frac{D_{\rm in}}{2} \\ 0 & \text{otherwise} \end{cases}, \tag{S3}$$

with $(k_y^a)^2 + (k_z^a)^2 = (n_{\rm in}\omega/c)^2$ for channel a, and $\{k_y^a\}$ are real-valued. The $\frac{1}{\sqrt{D_{\rm in}}}\frac{1}{\sqrt{k_z^a}}$ prefactors are used to normalize the longitudinal flux. The incident flux in z direction is then proportional to

$$\mathrm{Im}\int_{-\infty}^{\infty} dy\ (E_x^{\rm in})^*\frac{\partial E_x^{\rm in}}{\partial z}\bigg|_{z=0} = \sum_a |v_a|^2 + \mathrm{Re}\sum_a\sum_{b\neq a} v_a^* v_b\sqrt{\frac{k_z^b}{k_z^a}}\frac{\sin\left[(k_y^b - k_y^a)D_{\rm in}/2\right]}{2(k_y^b - k_y^a)D_{\rm in}}, \tag{S4}$$

where the summation only includes propagating channels (with real-valued k_z^a) as the evanescent ones do not carry flux.

Based on the Nyquist-Shannon sampling theorem [6], a discrete sampling of the momentum space with spacing $2\pi/D_{\rm in}$ is sufficient to represent a band-limited function with bandwidth $D_{\rm in}$ in real space. Therefore, we consider input wave numbers

$$\{k_y^a\} = \left\{a\frac{2\pi}{D_{\rm in}} \text{ such that } a \in \mathbb{Z} \text{ and } |k_y^a| < \frac{\omega}{c}\sin\left(\frac{\rm FOV}{2}\right)\right\}, \tag{S5}$$

limited to within the FOV of interest. Note that $\sin\frac{\rm FOV}{2} = n_{\rm in}\sin\frac{{\rm FOV}_{\rm in}}{2}$. With this choice, the different basis functions of Eq. (S3) are orthogonal, and the cross terms in Eq. (S4) drop out.

Similarly, the transmitted field $E_x(y, z \gtrsim h)$ near the output aperture can be expanded in a flux-orthogonal basis of truncated plane waves

$$E_x(y, z \gtrsim h) = \sum_b u_b g_b(y,z), \tag{S6}$$

where

$$g_b(y,z) = \begin{cases} \frac{1}{\sqrt{D_{\rm out}}}\frac{1}{\sqrt{k_z^b}} e^{i[k_y^b y + k_z^b(z-h)]} & \text{for } |y| < \frac{D_{\rm out}}{2} \\ 0 & \text{otherwise} \end{cases}, \tag{S7}$$

with

$$\{k_y^b\} = \left\{b\frac{2\pi}{D_{\rm out}} \text{ such that } b \in \mathbb{Z} \text{ and } |k_y^b| < \frac{\omega}{c}n_{\rm out}\right\}. \tag{S8}$$

and $(k_y^b)^2 + (k_z^b)^2 = (n_{\rm out}\omega/c)^2$. Note that the transmitted field can also have evanescent components, but here we only consider contributions from the propagating ones.

B. Transmission matrix in angular basis

Consider incident wave from a fixed angle

$$E_x^a(y', z=0) = f_a(y', z=0), \tag{S9}$$

so $v_{a'} = \delta_{aa'}$. Following Fig. 3 of the main text, the transmitted field for an ideal lens is

$$E_x^a(y, z=h) = \begin{cases} A(\theta_{\rm in})\frac{e^{i\phi_{\rm out}^{\rm ideal}(y,\theta_{\rm in})}}{[f^2+(y-y_{\rm f})^2]^{\frac{1}{4}}} & \text{for } |y| < D_{\rm out}/2 \\ 0 & \text{otherwise} \end{cases}, \tag{S10}$$

which we can project onto the preceding basis $\{g_b\}$ to get

$$u_b = \sqrt{\frac{k_z^b}{D_{\rm out}}}\int_{-\frac{D_{\rm out}}{2}}^{\frac{D_{\rm out}}{2}} E_x^a(y, z=h)e^{-ik_y^b y}dy. \tag{S11}$$

Since $u_b = \sum_{a'} t_{ba'}v_{a'} = t_{ba}$, this gives the a-th column of the transmission matrix in angular basis. To summarize, we have

$$\begin{aligned} t_{ba} &= \sqrt{\frac{k_z^b}{D_{\rm out}}}\int_{-\frac{D_{\rm out}}{2}}^{\frac{D_{\rm out}}{2}} E_x^a(y, z=h)e^{-ik_y^b y}dy, \\ \text{with } (k_y^a)^2 + (k_z^a)^2 &= (\frac{\omega}{c}n_{\rm in})^2,\ k_y^a = a\frac{2\pi}{D_{\rm in}},\ a \in \mathbb{Z},\ |k_y^a| < \frac{\omega}{c}\sin\left(\frac{\rm FOV}{2}\right) \\ (k_y^b)^2 + (k_z^b)^2 &= (\frac{\omega}{c}n_{\rm out})^2,\ k_y^b = b\frac{2\pi}{D_{\rm out}},\ b \in \mathbb{Z},\ |k_y^b| < \frac{\omega}{c}n_{\rm out}. \end{aligned} \tag{S12}$$

The normalization constant $A(\theta_{\rm in})$ can be determined from flux conservation, $\sum_b |t_{ba}|^2 = 1$, for an ideal lens with unity transmission.

To evaluate the transmission matrix in practice, we approximate the continuous integration over y in Eq. (S12) and in the evaluation of the inverse participation ratio (IPR) [Eq. (7) of the main text] with a discrete summation with spacing Δy. To determine the resolution to use, we use $\Delta y = \lambda/4$ and $\Delta y = \lambda/20$ to evaluate the maximal lateral spreading $\Delta W_{\rm max}$ for $D_{\rm out} = 200\lambda$ or 900λ, NA = 0.2 or 0.8, FOV = 40° or 160°. The relative difference of $\Delta W_{\rm max}$ between the two choices of Δy is smaller than 5% in all cases, so we use $\Delta y = \lambda/4$ in the following.

Eq. (S12) approximated with a discrete summation can be evaluated efficiently using fast Fourier transform (FFT),

$$t_{ba} \approx \Delta y\sqrt{\frac{k_z^b}{D_{\rm out}}}e^{-ik_y^b(-\frac{D_{\rm out}}{2}+\frac{\Delta y}{2})}\sum_{n=0}^{N-1} E_x^a(y_n, z=h)e^{-i\frac{2\pi}{N}bn}, \tag{S13}$$

where $N \equiv D_{\rm out}/\Delta y \in \mathbb{Z}$ is the number of segments the interval $y \in [-D_{\rm out}/2, D_{\rm out}/2]$ is discretized into, and $\{y_n \equiv -\frac{D_{\rm out}}{2} + \frac{\Delta y}{2} + n\Delta y\}$ are the centers of those segments. This produces N rows of the transmission matrix with $0 \le b \le N-1$; we cyclically rearrange the output index b and keep the propagating channels within $|k_y^b| < n_{\rm out}\omega/c$.

C. Transmission matrix in spatial basis

We define the transmission matrix in spatial basis, $t(y, y')$, by

$$E_x(y, z = h) \triangleq \int_{-\infty}^{\infty} t(y, y') E_x^{\rm in}(y', z = 0)\, dy', \tag{S14}$$

where only propagating waves within the input and output apertures are kept in the incident field $E_x^{\rm in}(y', z = 0)$ and in the transmitted field $E_x(y, z = h)$.

From Eqs. (S6,S7) with $u_b = \sum_a t_{ba} v_a$ and $v_a = \sqrt{\frac{k_z^a}{D_{\rm in}}} \int_{-\frac{D_{\rm in}}{2}}^{\frac{D_{\rm in}}{2}} E_x^{\rm in}(y', z = 0) e^{-ik_y^a y'} dy'$, we have

$$E_x(y, z = h) = \frac{1}{\sqrt{D_{\rm in} D_{\rm out}}} \sum_b \sum_a \sqrt{\frac{k_z^a}{k_z^b}} e^{ik_y^b y} t_{ba} \int_{-\frac{D_{\rm in}}{2}}^{\frac{D_{\rm in}}{2}} E_x^{\rm in}(y', z = 0) e^{-ik_y^a y'}\, dy' \tag{S15}$$

when $|y| < D_{\rm out}/2$. Comparing Eqs. (S14)(S15), we obtain the spatial transmission matrix

$$t(y, y') = \begin{cases} \frac{1}{\sqrt{D_{\rm in} D_{\rm out}}} \sum_b \sum_a \sqrt{\frac{k_z^a}{k_z^b}} e^{ik_y^b y} t_{ba} e^{-ik_y^a y'} & \text{when } |y| < D_{\rm out}/2 \text{ and } |y'| < D_{\rm in}/2 \\ 0 & \text{otherwise} \end{cases}. \tag{S16}$$

In Eq. (S16), the spatial coordinates y and y' are both continuous variables, which is redundant from the information point of view. Since the transmission matrix has a bandwidth of $|k_y| < (\omega/c)n_{\rm out}$ in the output and a bandwidth of $|k_y'| < (\omega/c)\sin({\rm FOV}/2)$ in the input, a discrete sampling of space with spacing $\Delta y = \frac{\lambda/n_{\rm out}}{2}$ in the output and spacing $\Delta y' = \frac{\lambda}{2\sin({\rm FOV}/2)}$ in the input should be sufficient, which is the Nyquist sampling rate. But accurate reconstruction would then require the Whittaker–Shannon interpolation. We skip the interpolation and simply use a finer resolution $\Delta y = \lambda/4$ in the output as mentioned in Sec. 2B. Since no integration is needed over the input y', we sample the input with spacing $\Delta y' = \frac{\lambda}{2\sin({\rm FOV}/2)}$. Eq. (S16) with discrete sampling can also be evaluated efficiently with FFT and inverse FFT.

Note that in order to make the transverse channels orthogonal, we imposed in Sec. 2A that k_y^a and k_y^b have spacing $2\pi/D_{\rm in}$ and $2\pi/D_{\rm out}$, which makes $E_x^{\rm in}(y', z = 0)$ and $E_x(y, z = h)$ periodic in y' and y with a period of $D_{\rm in}$ and $D_{\rm out}$ respectively. Such an artificial periodic boundary leads to periodic replications in the spatial transmission matrix, as shown in Fig. S5, which makes the lateral spreading inaccurate for y' near the two edges. To remove this unphysical periodic replication while maintaining orthogonality of the basis, we choose a $D_{\rm in}$ that is larger than $D_{\rm out}$ (specifically, $D_{\rm in} = 5D_{\rm out}$). Such choice does not affect the conclusion of this study since the angular variation of the phase shift (and subsequently $\Delta W_{\rm max}$ and the minimal thickness) depends on $D_{\rm out}$, not $D_{\rm in}$.

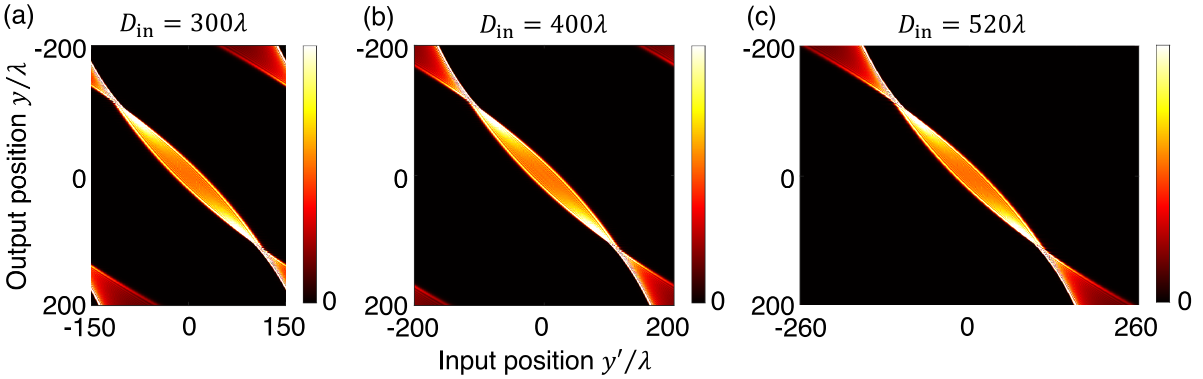

Fig. S5. Spatial transmission matrix $|t(y, y')|^2$ of an ideal wide-FOV lens with different $D_{\rm in}$. As $D_{\rm in}$ increases, the periodic replication goes away. Lens parameters: diameter $D_{\rm out} = 400\lambda$, NA = 0.45, FOV = 80°, with $\psi(\theta_{\rm in}) = \psi_0(\theta_{\rm in})$ and $y_{\rm f}(\theta_{\rm in}) = f\tan\theta_{\rm in}$.

3. OPTIMIZATION OF GLOBAL PHASE

The function $\psi(\theta_{\rm in})$ in Eq. (11) of the main text does not affect focusing quality, so we use it as a free parameter to minimize the maximal variation of $\Delta\phi_{\rm ideal}(y, \theta_{\rm in})$ with respect to $\theta_{\rm in}$, which minimizes $\Delta W_{\rm max}$ and the associated thickness bound.

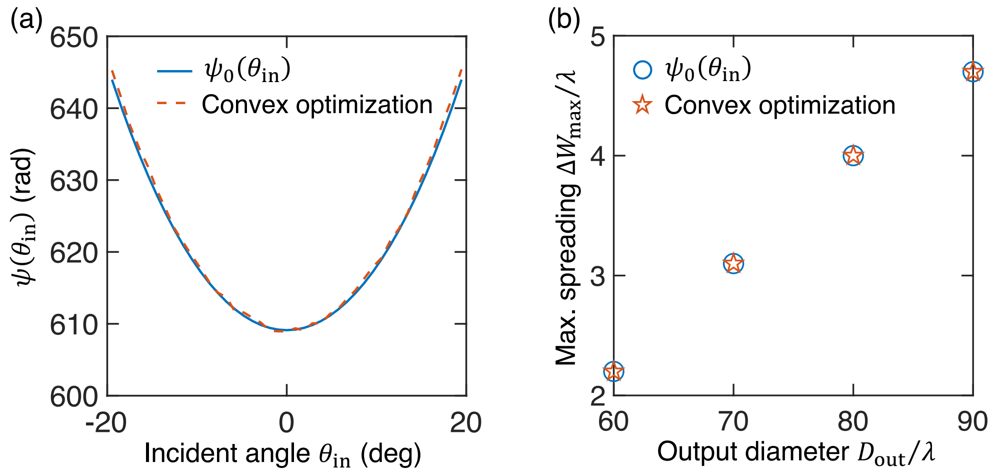

Fig. S6. Optimization with respect to $\psi(\theta_{\text{in}})$. (a) $\psi_0(\theta_{\text{in}})$ in Eq. (S17) and the $\psi(\theta_{\text{in}})$ from optimizing Eq. (S18). Lens parameters: diameter $D_{\text{out}} = 60\lambda$, NA $= 0.3$, FOV $= 40°$, $y_{\text{f}}(\theta_{\text{in}}) = f \tan\theta_{\text{in}}$. (b) The maximal lateral spreading ΔW_{max} as a function of the output diameter D_{out} after optimizing $\psi(\theta_{\text{in}})$.

As described in the main text, a sensible choice is

$$\psi(\theta_{\text{in}}) = \frac{2\pi}{\lambda}\left\langle \sqrt{f^2 + [y - y_{\text{f}}(\theta_{\text{in}})]^2} + y\sin\theta_{\text{in}} \right\rangle_y \equiv \psi_0(\theta_{\text{in}}), \tag{S17}$$

where $\langle\cdots\rangle_y$ denotes averaging over y within $|y| < D_{\text{out}}/2$.

To assess the performance of the $\psi_0(\theta_{\text{in}})$ above, we carry out a numerical optimization where $\psi(\theta_{\text{in}})$ is varied to minimize the maximal phase-shift difference between all possible pairs of incident angles across all positions:

$$\underset{\psi(\theta_{\text{in}})}{\text{argmin}} \; \max_{y,\theta^i_{\text{in}},\theta^j_{\text{in}}} |\Delta\phi_{\text{ideal}}(y,\theta^i_{\text{in}};\psi) - \Delta\phi_{\text{ideal}}(y,\theta^j_{\text{in}};\psi)|^2, \tag{S18}$$

where θ^i_{in} and θ^j_{in} represent different incident angles with $|\theta^{i,j}_{\text{in}}| < \text{FOV}/2$, $|y| < D_{\text{out}}/2$, and

$$\begin{aligned}\Delta\phi_{\text{ideal}}(y,\theta_{\text{in}};\psi) &= \phi^{\text{ideal}}_{\text{out}}(y,\theta_{\text{in}}) - \phi_{\text{in}}(y,\theta_{\text{in}}) \\ &= \psi(\theta_{\text{in}}) - \frac{2\pi}{\lambda}\left[\sqrt{f^2 + [y - y_{\text{f}}(\theta_{\text{in}})]^2} + y\sin\theta_{\text{in}}\right].\end{aligned} \tag{S19}$$

This is a convex optimization problem [7] because $\Delta\phi_{\text{ideal}}(y,\theta_{\text{in}};\psi)$ is a linear function of ψ. We use CVX [8, 9], a package for specifying and solving convex programming problems, to find the global optimum of Eq. (S18).

The global optimum of $\psi(\theta_{\text{in}})$ is in close agreement with the $\psi_0(\theta_{\text{in}})$ in Eq. (S17), as shown in Fig. S6(a). While there are small differences among the two, such differences have no noticeable effect on the resulting maximal lateral spreading ΔW_{max}, as shown in Fig. S6(b). Therefore, in the following, we use $\psi = \psi_0$ in Eq. (S17) as the global phase.

4. FOV DEPENDENCE (ANIMATION)

Supplementary Video 1 shows how the ideal transmission matrix (in both bases) and the phase-shift profiles evolve as the FOV increases. Figure S7 provides the animation caption and shows one frame of the animation. Increasing the FOV leads to an increased phase-shift variation among incident angles, which widens the diagonal of the spatial transmission matrix.

5. OUTPUT PROFILES AND OUTPUT WIDTHS

Figure S8 shows the middle column of the spatial transmission matrix for ideal wide-FOV lenses with different FOVs. With a small FOV, the output profiles are reasonably close to being rectangular, and the inverse participation ratio (IPR) coincides with the full width at half maximum (FWHM). With a large FOV, the output profiles develop two strong peaks, and the IPR underestimates the output width.

6. COMPREHENSIVE DATA FOR DEPENDENCE ON LENS PARAMETERS

Figure 5(a,b) of the main text plots the optimized maximal lateral spreading ΔW_{max} for NA $= 0.7$ and output diameter $D_{\text{out}} = 300\lambda$ respectively, with varying FOV. Figures S9–S10 plot ΔW_{max} for other NA and other D_{out}.

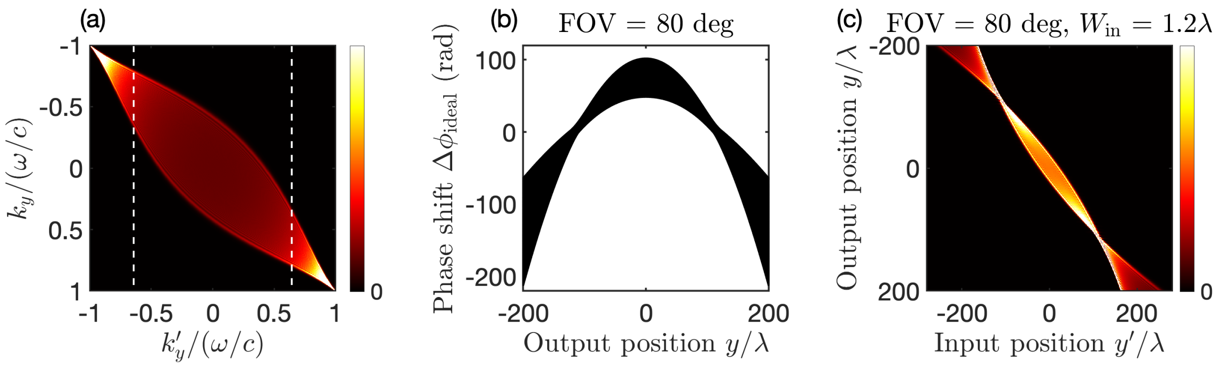

Fig. S7. One frame of Supplementary Video 1, which shows how the transmission matrix of an ideal lens evolves with the FOV. (a) The angular transmission matrix $|t(k_y, k'_y)|^2$; white dashed lines show the boundary given by the FOV. (b) Phase-shift profiles across incident angles within the FOV. (c) The corresponding spatial transmission matrix $|t(y, y')|^2$ at the given FOV. Lens parameters: diameter $D_{\text{out}} = 400\lambda$, NA = 0.45, with $\psi(\theta_{\text{in}}) = \psi_0(\theta_{\text{in}})$ and $y_{\text{f}}(\theta_{\text{in}}) = f \tan\theta_{\text{in}}$.

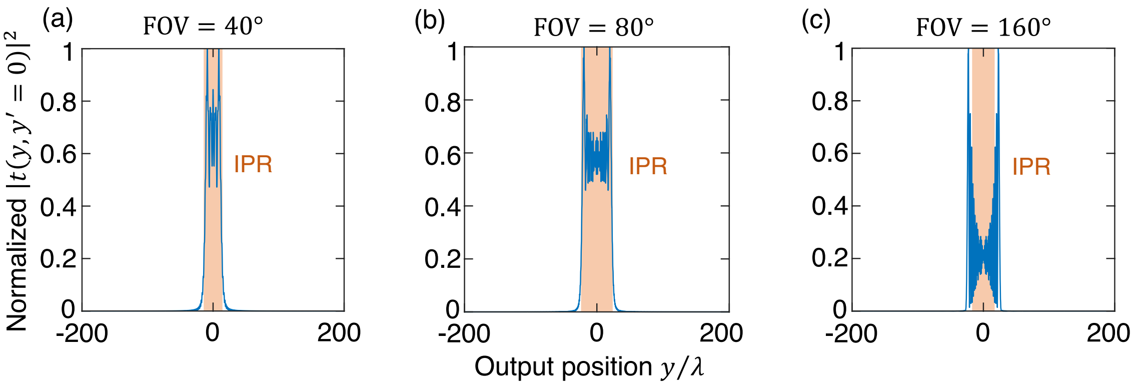

Fig. S8. Output profiles of the ideal spatial transmission matrix (blue lines), with the widths defined by the inverse participation ratio (IPR) indicated with orange shadings. Lens parameters: $D_{\text{out}} = 400\lambda$, NA = 0.45, with $\psi(\theta_{\text{in}}) = \psi_0(\theta_{\text{in}})$ and $y_{\text{f}}(\theta_{\text{in}}) = f \tan\theta_{\text{in}}$.

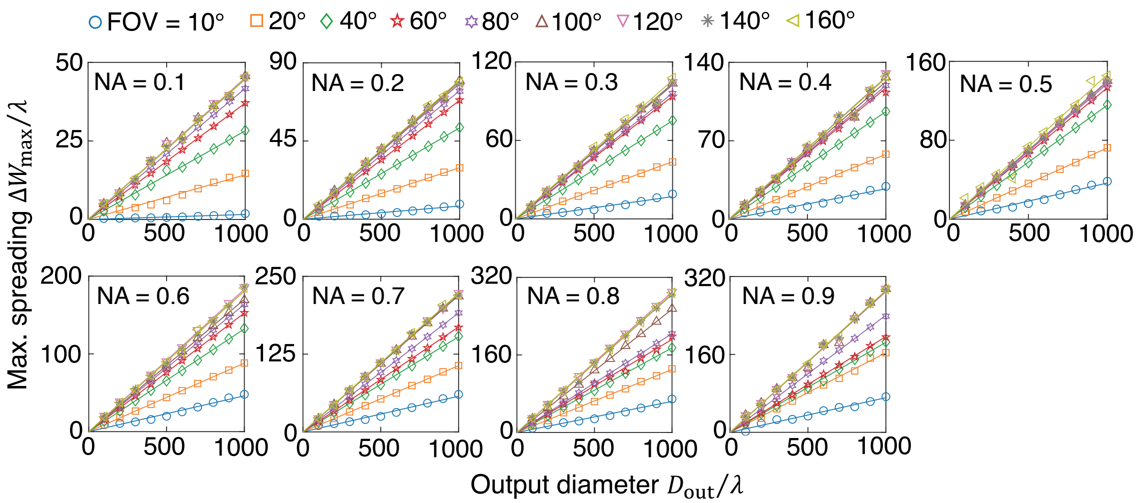

Fig. S9. Optimized maximal lateral spreading as a function of the output diameter D_{out} for different NA and FOV.

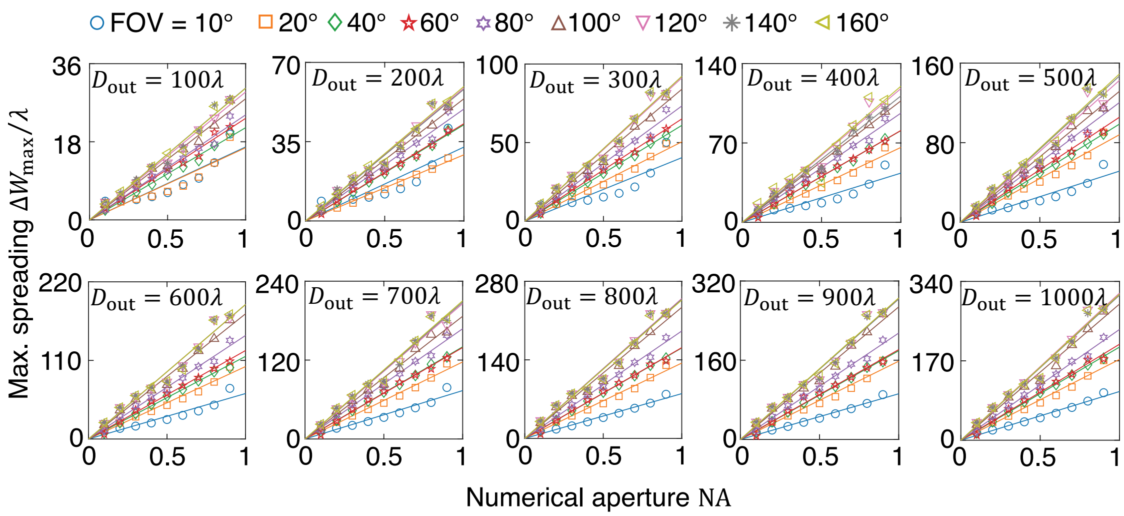

Fig. S10. Optimized maximal lateral spreading as a function of the numerical aperture NA for different lens diameter D_{out} and FOV.

REFERENCES

1. E. Hecht, “Chapter 5.2: Lenses,” in *Optics, 5ed,* (Pearson Education Limited, 2017).
2. F. Aieta, P. Genevet, M. A. Kats, N. Yu, R. Blanchard, Z. Gaburro, and F. Capasso, “Aberration-free ultrathin flat lenses and axicons at telecom wavelengths based on plasmonic metasurfaces,” Nano Lett. **12**, 4932–4936 (2012).
3. M. Pu, X. Li, Y. Guo, X. Ma, and X. Luo, “Nanoapertures with ordered rotations: symmetry transformation and wide-angle flat lensing,” Opt. Express **25**, 31471–31477 (2017).
4. A. Martins, K. Li, J. Li, H. Liang, D. Conteduca, B.-H. V. Borges, T. F. Krauss, and E. R. Martins, “On metalenses with arbitrarily wide field of view,” ACS Photonics **7**, 2073–2079 (2020).
5. E. Lassalle, T. W. Mass, D. Eschimese, A. V. Baranikov, E. Khaidarov, S. Li, R. Paniagua-Dominguez, and A. I. Kuznetsov, “Imaging properties of large field-of-view quadratic metalenses and their applications to fingerprint detection,” ACS Photonics **8**, 1457–1468 (2021).
6. H. Landau, “Sampling, data transmission, and the Nyquist rate,” Proc. IEEE **55**, 1701–1706 (1967).
7. S. Boyd and L. Vandenberghe, *Convex optimization* (Cambridge university press, 2004).
8. M. Grant and S. Boyd, “CVX: Matlab software for disciplined convex programming, version 2.1,” http://cvxr.com/cvx (2014).
9. M. Grant and S. Boyd, “Graph implementations for nonsmooth convex programs,” in *Recent Advances in Learning and Control,* V. Blondel, S. Boyd, and H. Kimura, eds. (Springer-Verlag Limited, 2008), Lecture Notes in Control and Information Sciences, pp. 95–110.
